# Effect of the developmental stage and tissue position on the expression and glycosylation of recombinant glycoprotein GA733-FcK in transgenic plants

**DOI:** 10.3389/fpls.2014.00778

**Published:** 2015-01-13

**Authors:** Chae-Yeon Lim, Kyung Jin Lee, Doo-Byoung Oh, Kisung Ko

**Affiliations:** ^1^Department of Medicine, Medical Research Institute, College of Medicine, Chung-Ang UniversitySeoul, South Korea; ^2^Biochemicals and Synthetic Biology Research Center, Korea Research Institute of Bioscience and BiotechnologyDaejeon, South Korea

**Keywords:** *N*-glycosylation, developmental stage, tissue position, transgenic plant, GA733-FcK, recombinant glycoprotein

## Abstract

The influence of developmental stage and position (top, middle, and base) of leaves and stem tissues on the expression and glycosylation pattern of a recombinant therapeutic protein -GA733-FcK- was observed in transgenic seedlings during a 16-week growth period. RNA expression gradually increased with age in the middle and basal leaves and decreased in top leaves after 14 weeks. The protein expression level at all leaf positions increased until 14 weeks and slightly decreased at 16 weeks; it was lower in yellow leaves than in green leaves. In stem, protein expression gradually decreased from the top to the base. The glycosylation patterns of GA733-FcK were analyzed from 10 to 16 weeks. The plant-specific glycans increased in the top leaves at 14 weeks, but only slightly changed in the middle and basal leaves. The structure of glycans varied with tissue position. The glycosylation level in the top and middle leaves increased until 12 and 14 weeks, respectively, and decreased thereafter, whereas it decreased in basal leaves until 14 weeks and increased at 16 weeks. In stem, all three sections showed high-mannose type glycan structures. The area size of the glycans was significantly higher in the top stem than in both the middle and basal stems, and it was smaller in yellow leaves than in green leaves. The glycan profiles were similar between green and yellow leaves until 16 weeks. Thus, biomass-harvesting time should be optimized to obtain recombinant therapeutic proteins with ideal glycan structure profiles.

## Introduction

Plants have been considered a promising bio-organism for production of highly valuable recombinant prophylactic and therapeutic proteins because of the overall cost effective production, post-translational adjustment, and glycosylation that is similar to that of mammalian cells (Rigano and Walmsley, 2005; Daniell et al., 2009). It is often claimed that production of highly valuable, plant-derived recombinant proteins in a plant needs only simple inputs (sunlight, water, and nutrients) that will enable the plant's rapid increase in biomass. Environmental factors that affect production of the plant biomass include temperature, light, salinity, drought, nutrition, insects, and pests (Criddle et al., 1997; Elbers et al., 2001; Jamal et al., 2013). The degree of recombinant gene expression varies in accordance with the increasing environmental factors such as temperature, day length, compost nitrogen content, nutrients, radiation, and plant density, which in turn affect the cultivation conditions of plants (Stevens et al., 2000; Elbers et al., 2001). Aging and position of tissues determine both the quality and quantity of recombinant protein produced in transgenic plants. Thus, the harvesting time and location of the high biomass tissues should be optimized to obtain recombinant therapeutic proteins with ideal glycan structure profiles.

In our previous study, the colorectal cancer vaccine candidate GA733-Fc recombinant protein was successfully expressed in a transgenic plant. GA733 is an epithelial cell adhesion molecule that is abundant in colorectal cancer cells. The extracellular domain of GA733 is often used as a target for cancer vaccination (Lu et al., 2012). A plant-derived recombinant GA733-Fc fused to KDEL endoplasmic reticulum (ER) retention signal was confirmed to have vaccine-like efficacy in animals. The antigen-antibody complex GA733-FcK may potentially have properties similar to the parental IgG, including enhanced efficacy of vaccination by targeting the vaccine to antigen-presenting cells, facilitated purification by the protein-A method, and increased half-life (Lu et al., 2012). To obtain such a highly valuable recombinant antigen-antibody complex proteins with high expression levels, better yields of plant biomass should be established in plant expression systems (Lo et al., 1998; Flanagan et al., 2007; Lu et al., 2012). However, how the tissue position and aging of a plant affect the glycan structure and protein expression level has not been investigated. Most of therapeutic proteins are glycoproteins, containing glycans with crucial roles in protein folding, therapeutic efficacy, *in vivo* half-lives, and immunogenicity (Lee et al., 2013). Glycoproteins produced from plants are not identical to the therapeutic proteins produced in human or mammalian cells, and plant-specific glycoproteins of xylose and fucose may induce an allergic reaction when administered to a human (Daniell et al., 2001, 2009; Tekoah et al., 2004). To reduce the possibility of an allergic reaction, the predominant expression system for therapeutic glycoproteins is retained in ER, which is the site of protein glycosylation, assembly, and folding (Sriraman et al., 2004). Thus, in this study, a variation of the GA733-FcK expression and its *N*-glycan structure were investigated in plant tissues at different locations and growth stage. The location and age of the tissues influence both the quality and quantity of recombinant proteins produced in transgenic plants (Stevens et al., 2000; Valdes et al., 2003a; Jamal et al., 2009). The objectives of this study were to investigate the effect of the developmental stage and position of plant leaves and tissues along the stem on expression and glycosylation of the recombinant GA733-FcK protein, and to determine the best harvesting time and tissue position for high protein expression and ideal glycosylation.

## Materials and methods

### Plant material

The GA733-FcK gene was cloned under the control of the enhanced cauliflower mosaic virus (CaMV) 35S promoter and tobacco etch viral 5′-leader sequence (TEV). Seedlings of transgenic tobacco (*Nicotiana tabacum*) line 303 seedlings highly expressing the tumor-associated antigen against GA733-FcK recombinant therapeutic proteins (Lu et al., 2012) were transplanted and grown in soil pots for 18 weeks in a greenhouse. Two hundred plants were used in the plant growth analysis. The leaf length was measured on the largest leaf, and plant height was measured from the base of the plant stem to the uppermost leaf. The position of leaves along the stem was divided into three equal sections: top, middle, and base, and leaf samples were collected from the top, middle, and base of the transgenic tobacco plant. Leaves were harvested at the age of 10, 12, 14, and 16 weeks after transplanting. Stem sections (top, middle, and base), including yellow and green leaves, were harvested at the age of 18 weeks.

### RNA isolation and quantitative real-time PCR analysis

The transcription level of GA733-FcK was quantified with a real-time (RT)-PCR. Total RNA was extracted from transgenic plant samples (top, middle, base) using an RNeasy Plant Mini Kit (Qiagen, Valencia, CA, USA) according to the manufacturer's protocol. At least 10 plants for each developmental stage and tissue positions were used for real-time PCR analysis. To remove the genomic DNA, 400 ng of total RNA was treated using a TURBO DNA-free kit (Ambion, Austin, TX, USA) in a reaction volume of 20 μL, and the isolated RNA samples were stored at −80°C until further use. Each RNA sample was used as a template in RT-PCR reactions performed using GoScript™ Reverse Transcription System (Promega, Madison, WI, USA) and GA733-FcK specific forward 5′-ATCTGGATCCTGGTCAAA-3′ and reverse primer 5′-CTCAGAGCAGGTTATTTC A-3′. PCR reactions (25 μL) contained 12.5 μL SYBR Green PCR Master Mix (Promega, Madison, WI, USA), 4 μL cDNA solution (equivalent to ~100 ng template), and primers at final concentrations of 1.0 μM. PCR was performed using a real-time PCR machine (Rotor-Gene Q, Qiagen) with the following cycling parameters: 5 min at 95°C, 5 s at 95°C, 10 s at 60°C, and 40 cycles of 5 s at 95°C and 10 s at 60°C. PCR reactions containing cDNA or “no template” control (NTC; containing only RNase-free water) were run in parallel for each template and primer combination. In qPCR analysis, the EF-1α gene involved in plant growth was used as a reference gene. Real-time PCR analysis was performed for more than 3 times.

### Immunoblot analysis

At least 10 plants for analysis each developmental stage and tissue positions were used for immunoblot blot. All samples were used with the same fresh weight of leaves and stems. Hundred milligrams of fresh leaves and stems (top, middle, and base) were homogenized in 350 μL 1 × PBS (137 mM NaCl, 10 mM Na_2_HPO_4_, 2.7 mM KCl, and 2 mM KH_2_PO_4_) to extract the total soluble proteins. A volume of 20 μL of extracted sample (100 mg of fresh leaf/300 μL) was mixed with 4 μL of loading buffer (1 M Tris-HCl, 50% glycerol, 10% SDS, 5% 2-mercaptoethanol, 0.1% bromophenol blue), loaded in 10% SDS-PAGE, and transferred to a nitrocellulose membrane (Millipore Corp., Billerica, MA, USA). Membranes were blocked with 5% skim milk (Sigma, St. Louis, MO, USA) in 1 × PBS-T buffer (1 × PBS plus 0.5% [v/v] Tween 20) for 2 h at room temperature. The blots were incubated for 1 h 30 min at room temperature with goat anti-human Fcγ recognizing the Fc portion of GA733-FcK. The protein bands were detected using “SuperSignal” chemiluminescence substrate (Pierce, Rockford, IL, USA) and visualized by exposing the membrane to an X-ray film (Fuji, Tokyo, Japan). The purified GA733-FcK was used as a positive control. Immunoblot analysis was performed for more than 3 times. The detected protein bands were digitized to an electronic image, and the band intensity was measured using Image J software (National Institutes of Health, Bethesda, MD). To quantify [ng/(mg of fresh leaf)] the amount of GA733-FcK in transgenic plants, the positive control (31.25, 62.5, 125, 250, and 500 ng) were loaded onto one SDS-PAGE gel (Supplemental Data Sheet 3). The intensity of each sample and standard was adjusted by subtracting background intensity from the measured intensity. A standard curve between the amount (ng) of the positive control and the measured image intensity of the standard was calculated using Image J software (National Institutes of Health, Bethesda, MD). The intensity value was used to estimate the amount of GA733-FcK proteins (ng/mg) from the linear regression equation derived from the standards.

### Purification of recombinant protein GA733-FcK from transgenic plants

At least 10 plants for each developmental stage and tissue positions were used for purification. For purification of plant GA733-FcK, tobacco plant leaves or stems were homogenized in an HR2094 aluminum blender (Philips, Seoul, Korea) on ice with extraction buffer (37.5 mM Tris-HCl pH 7.5, 50 mM NaCl, 15 mM EDTA, 75 mM sodium citrate, and 0.2% sodium thiosulfate). After centrifugation at 8800× *g* for 30 min at 4°C, the suspension was filtered through a Miracloth (Biosciences, La Jolla, CA, USA) and its pH was adjusted to 5.1 by addition of acetic acid pH 2.4. The solution was centrifuged at 10,200× *g* for 30 min at 4°C. The pH of the solution was brought back to 7.0 by addition of 3 M Tris-HCl, and ammonium sulfate was added to 8% saturation. After centrifugation at 8,800× *g* for 30 min at 4°C, the precipitate was discarded and ammonium sulfate was added to the supernatant to 40% saturation. After an overnight incubation at 4°C, the solution was again centrifuged, the pellet was resuspended in extraction buffer to 1/10 of the original volume, and the final solution was then centrifuged at 10,200× *g* for 30 min at 4°C. The supernatant of extracted sample was then filtered through a 0.45-mm filter and loaded onto a HiTrap Protein G column (Pharmacia, Uppsala, Sweden). Soluble protein extract was applied to a protein G column (GE Healthcare, Piscataway, NJ, USA). Elutes of plant-derived recombinant GA733-Fc protein were dialyzed against 1 × PBS buffer. Aliquots were frozen in liquid nitrogen and stored at −80°C for glycosylation analysis (Supplemental Data Sheet 2).

### Glycan analysis

The purified samples were incubated twice with 2 μL of pepsin in an incubator at 37°C for 12 h to digest the protein into glycopeptides. The digested glycopeptides were collected using a C18 sep-pak cartridge (Waters, Lexington, MA, USA). Briefly, samples were passed through a C18 sep-pak cartridge and washed with 5% acetic acid to remove contaminants such as salts and free sugar. The fraction containing peptides and glycopeptides was eluted in a series of solutions with 20% iso-propanol in 5% acetic acid, 40% iso-propanol in 5% acetic acid, and 100% iso-propanol, and the eluted fractions were dried in a speed vacuum system. PNGase A (Roche, Basel, Switzerland) was added to the samples to release *N*-glycans, and the samples were incubated overnight at 37°C. The released *N*-glycans were purified from the samples by using a graphitized carbon resin from Carbograph (Alltech, Lexington, MA, USA).

### HPLC analysis of AB labeled glycans

For detection in HPLC, purified glycans were aminobenzamide (AB)-labeled using previously described methods with slight modifications (Bigge et al., 1995). Briefly, the labeling reagent was freshly prepared by dissolving 6 mg of AB in 100 μL 30% (v/v) acetic acid in a dimethyl sulfoxide solution containing 1 M of sodium cyanoborohydride. Then, each dried glycan sample was dissolved in 5 μL of a labeling reagent and incubated overnight in a tightly capped tube at 37°C. The mixture was diluted with 1 mL of 96% (v/v) acetonitrile in water, and excess labeling reagent was removed using a cyano cartridge (Agilent Technologies, Santa Clara, CA, USA) following the manufacturer's instructions. Purified AB-labeled glycans were separated on a TSK amide-80 (5 μm, 4.6 mm × 250 mm) column (Tosoh Bioscience, Prussia, PA, USA) using the previously described HPLC systems with a fluorescence detector (330 nm excitation and 425 nm emission). Separations were achieved at a flow rate of 1.0 mL/min using a mixture of solvent A (100% acetonitrile) and solvent B (50 mM ammonium formate, pH 4.4). After the column was equilibrated with 30% solvent B, the sample was injected and then eluted by a linear gradient to 45% of solvent B for 60 min. HPLC analysis was performed for more than 3 times.

### Statistical analysis

All data were presented as the mean ± SD. Comparisons of multiple groups were performed by One-Way analysis of variance (ANOVA) and Two-Way ANOVA, followed by pairwise comparisons with a Bonferroni *post-hoc* test. Differences were considered statistically significant at *p* < 0.05. All data were analyzed using the GraphPad Prism software, version 5.00 (Graph-Pad software, San Diego, CA).

## Results

### Growth patterns of plant height and leaf length

**Figure 1:**
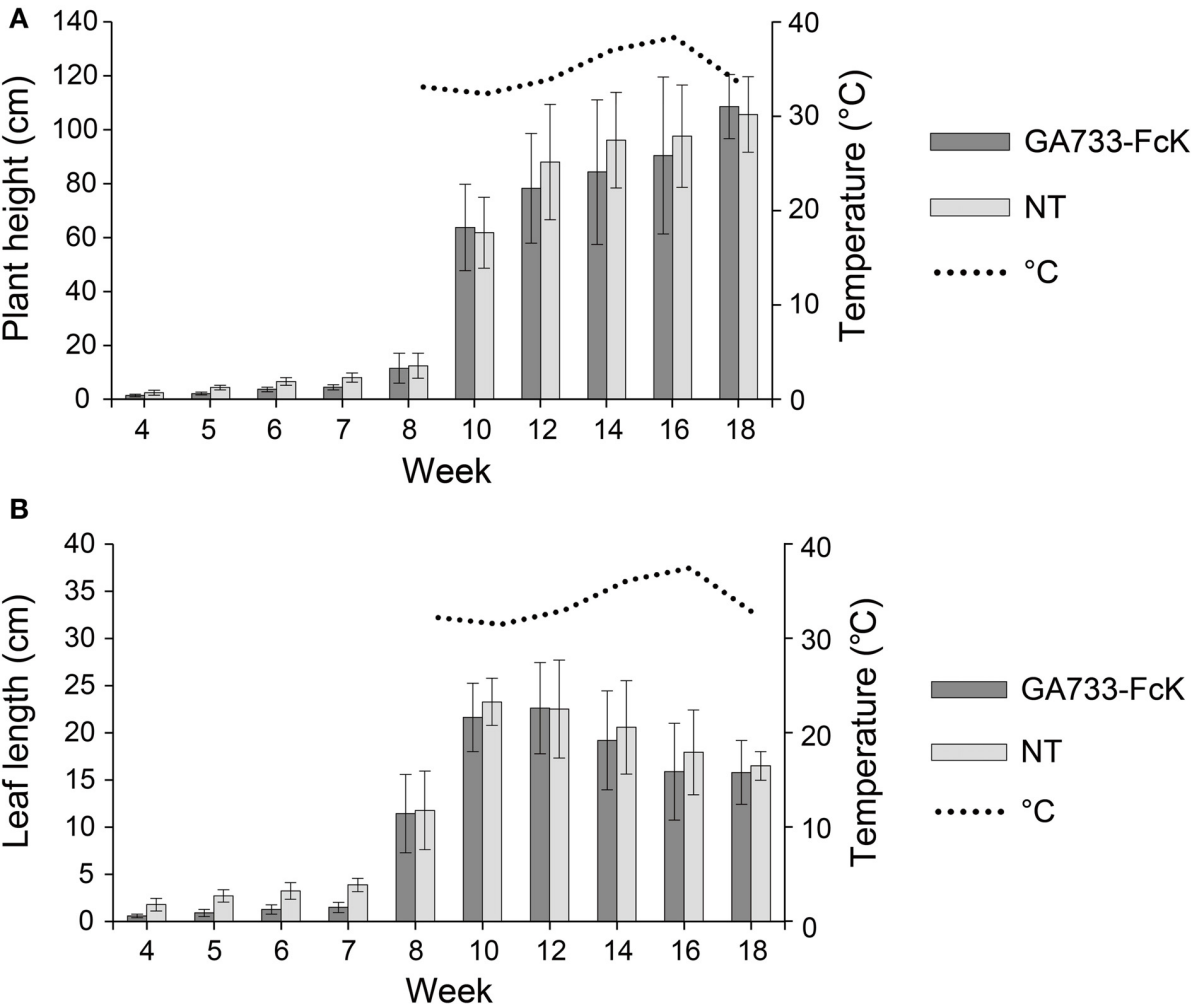
**Plant growth over time of GA733-FcK transgenic tobacco and non-transgenic tobacco**. **(A)** Averages of the height. **(B)** Averages of the leaf length.

To investigate the change in plant biomass expressed through plant height and leaf length, and the variation in expression of GA733-FcK during the growth period under greenhouse conditions, F_3_ seedlings of transgenic plant expressing GA733-FcK were transplanted into soil pots and grown in a greenhouse for 18 weeks. There was no significant difference in plant height and leaf length between transgenic plants expressing GA733-FcK and non-transgenic plants (Figure 1). The plant height increased at fast pace for approximately 8 weeks, and it continued increasing until 18 weeks (Figure 1). The leaf length rapidly increased from 7 to 10 weeks and then slowly decreased. At 14 weeks, the plants started to blossom, and at 18 weeks of fertilization and seed-pod formation occurred. Overall, the growth patterns between transgenic and non-transgenic plants were similar.

### Effect of plant growth stage on transcriptional level of GA733-FcK gene

**Figure 2:**
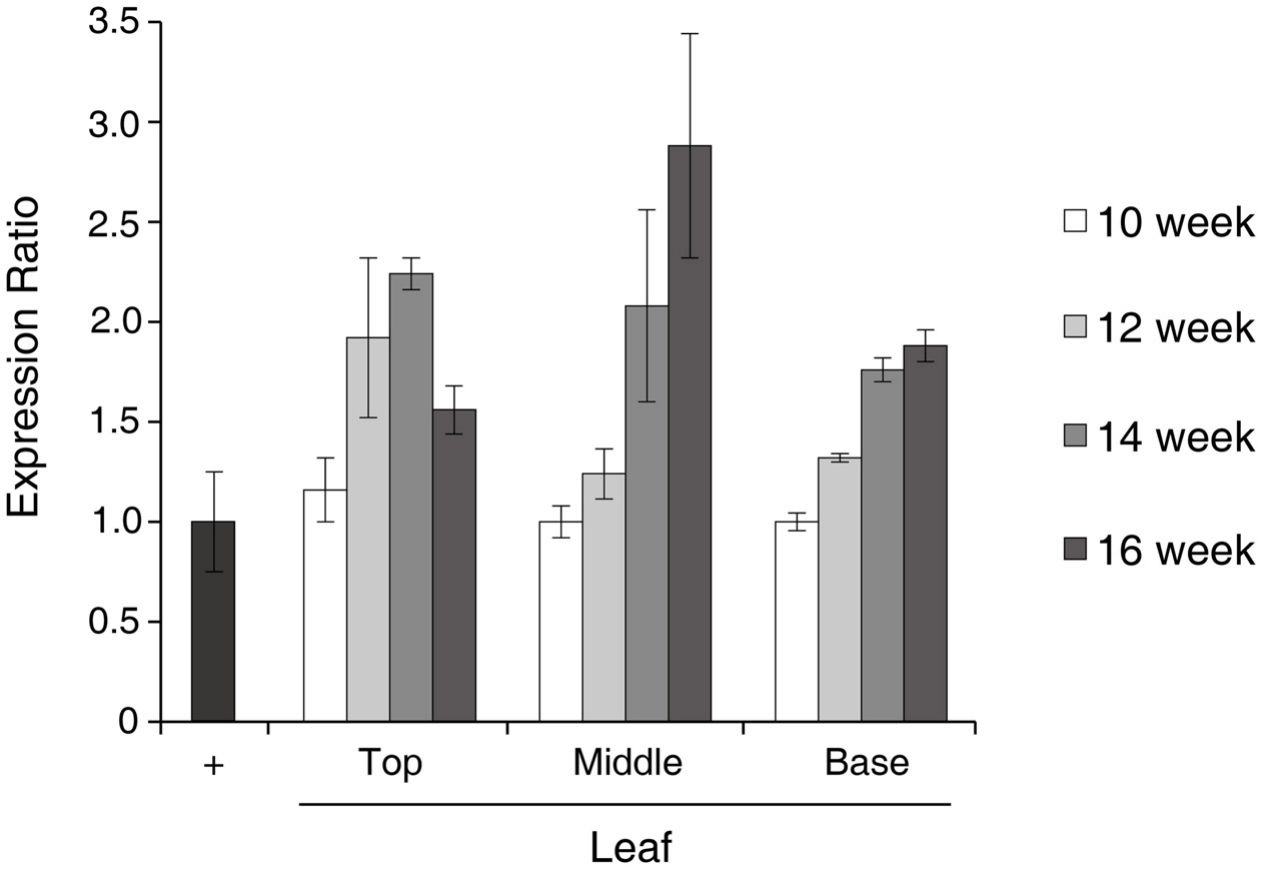
**Characterization of GA733-FcK RNA expression patterns by real-time PCR**. Relative expression levels of GA733-FcK with aging and at different positions of leaf tissues by real-time PCR using cDNAs equivalent to 400 ng of total RNA. Positive control is the isolated RNA of GA733-FcK transgenic plant leaf (40 ng).

Real-time PCR was used to determine the transcriptional level of the GA733-FcK gene in samples of top (T), middle (M), and basal (B) leaves harvested from tobacco plants at 10, 12, 14, and 16 weeks after transplanting (Figure 2). In the top leaves, the expression ratio increased until 14 weeks followed by a decrease. In the middle and base leaves, the expression ratio continuously increased until 16 weeks. However, the basal leaves did not show rapid increase in expression ratio compared to the middle leaves.

### Effect of plant growth stage on GA733-FcK protein level in leaves

**Figure 3:**
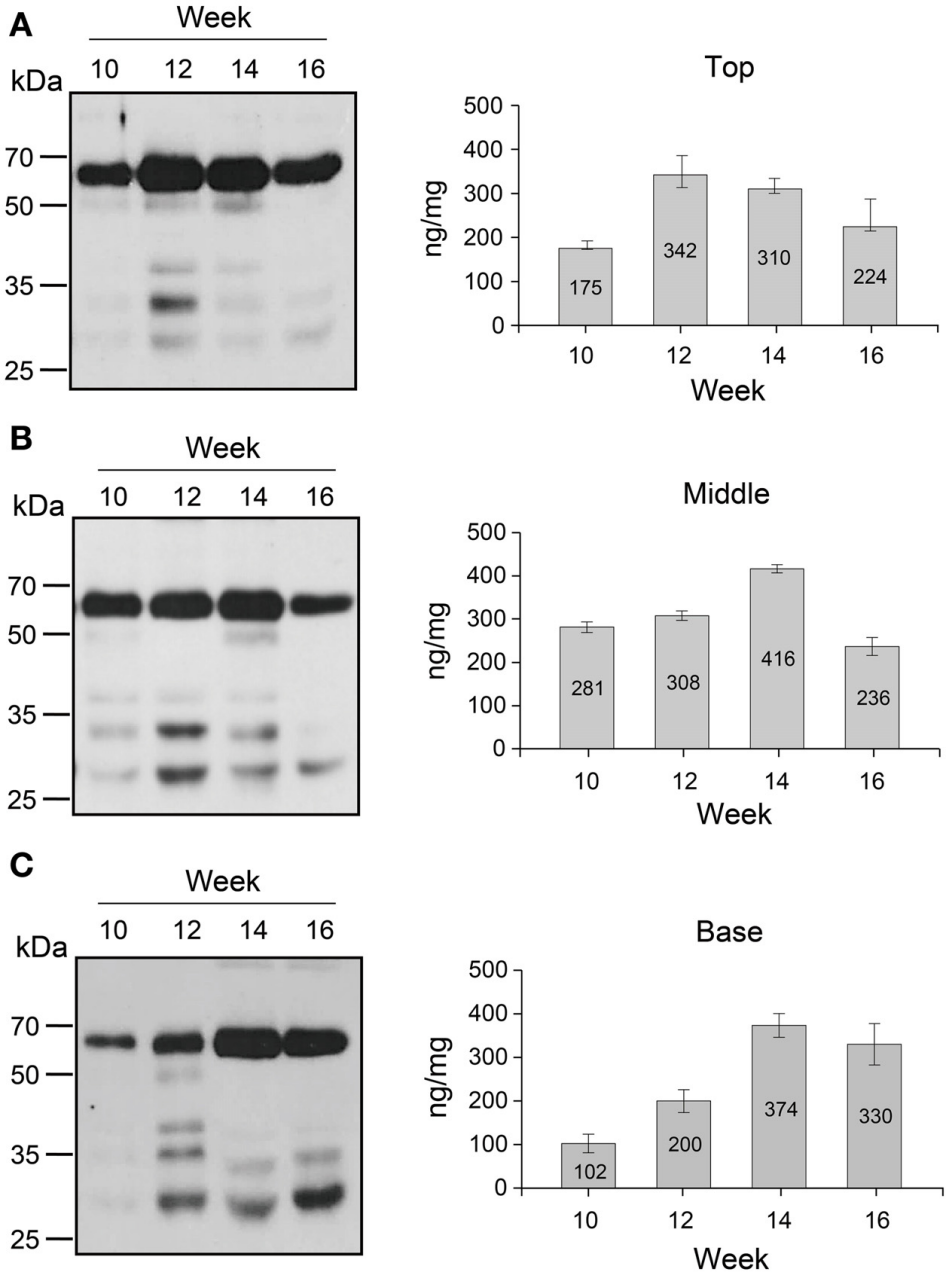
**Western blot analysis**. **(A)** Western blot of the top leaf expression. **(B)** Western blot of the middle leaf expression. **(C)** Western blot of the basal leaf expression. Leaf samples from GA733-FcK transgenic plants were ground with extraction buffer to confirm the protein expression levels. The GA733-FcK proteins were detected by mouse anti-human Fcγ antibody. The graph shows relative quantitation.

The changes in GA733-FcK protein levels in top, middle, and basal leaves at 10, 12, 14, and 16 weeks after transplanting were investigated by western blotting (Figure 3). In the top leaves, the GA733-FcK protein level increased until 12 weeks (342 ng/mg fresh weight; FW) and decreased afterwards, whereas in the middle and basal leaves, the protein levels increased until 14 weeks (416 and 374 ng/mg FW, respectively). The middle leaves showed the highest GA733-FcK protein levels at 10 weeks (281 ng/mg FW) and 14 weeks (416 ng/mg FW). However, at 14 weeks, basal leaves showed the highest protein level. The band density of non-specific proteins below 50 kDa was lower in the top leaves than in the others. At 16 weeks, the basal leaves showed relatively stronger non-specific band density than the top and middle leaves. The amount of detected GA733-FcK protein on each lane was relatively quantified with comparison of the known amount of the positive control. The positive controls were not shown on the Figure 3 in this manuscript.

### Expression level of GA733-FcK in stems and senescent leaves at 18 weeks

**Figure 4:**
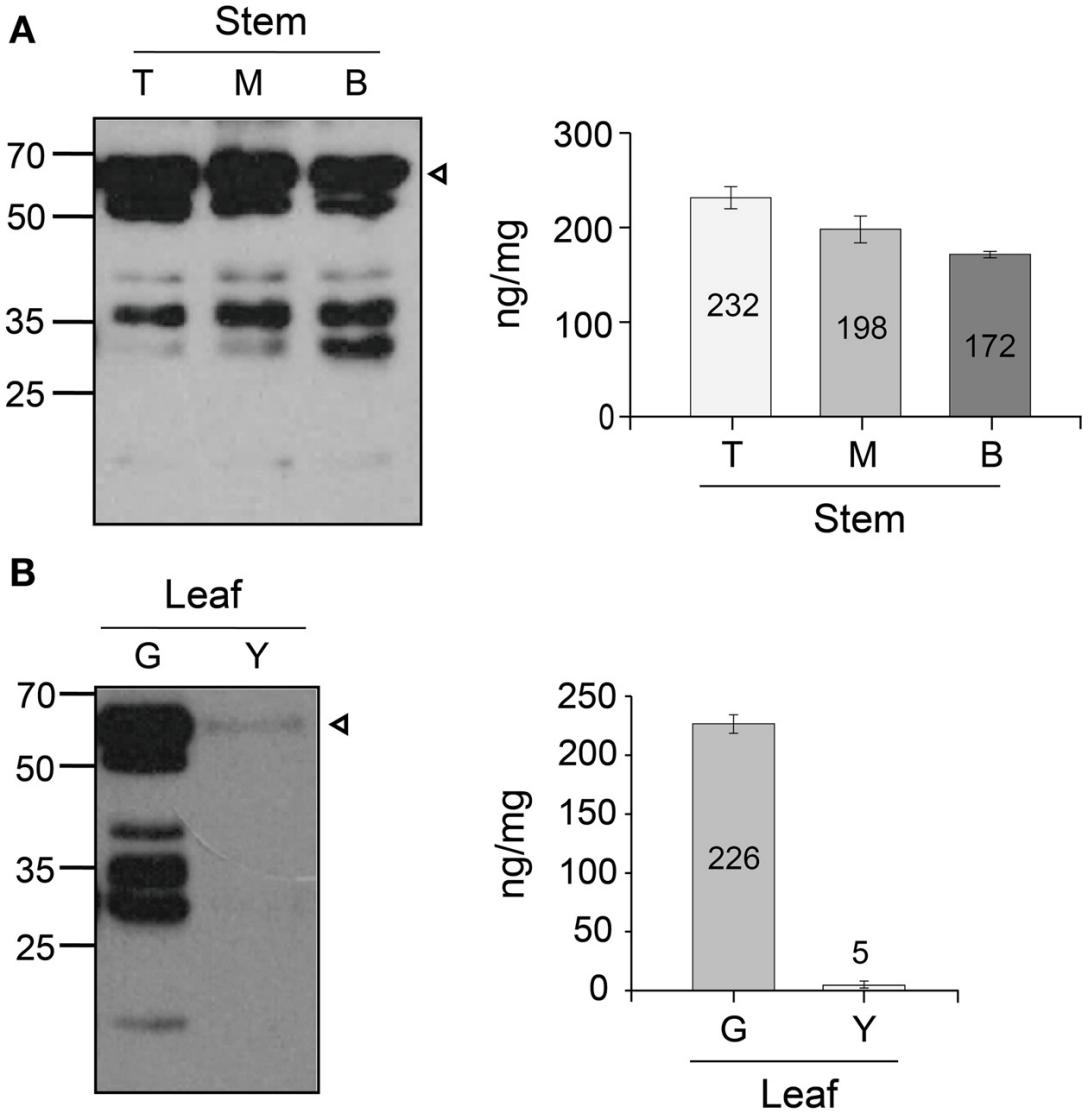
**Immunoblots showing protein GA733-FcK band of yellow and green leaves and stem**. **(A)** Western blot of the stem expression. **(B)** Western blot of the green leaf and yellow leaf expression. Leaf samples from GA733-FcK transgenic plants were ground with extraction buffer to confirm the protein expression levels. The GA733-FcK proteins were detected by mouse anti-human Fcγ antibody. The graph shows relative quantitation.

The different expression patterns of top, middle, and basal stem sections harvested from plants at 18 weeks were analyzed by using the densitometry analysis (Figure 4A). The protein level (ng/mg FW) was calculated by using the standard curve between the band density of the purified GA733-FcK and their known protein concentrations. The protein level decreased from the top to the base of the stem (top [232 ng/mg FW], middle [198 ng/mg FW], and base [172 ng/mg FW]) (Figure 4A). The non-specific bands were observed below the GA733-FcK band in all stem samples. The smallest band (~30 kDa) (Figure 4A) showed stronger density at the base of the stem than in other sections of the stem. In addition, the differential expression patterns of the GA733-FcK protein levels were determined between green and yellow leaves harvested from plants at 18 weeks (Figure 4B). The GA733-FcK protein band was observed with several non-specific bands, which are speculated to be proteolytically degraded protein bands. However, in yellow leaves, the GA733-FcK protein band was barely observed. Densitometry quantitative analysis showed that the GA733-FcK protein concentration (5 ng/mg FW) in yellow leaves was 45 times lower than that (266 ng/mg FW) in the green leaves.

### Glycosylation variation of GA733-FcK at different tissue positions and different growth stage

**Figure 5:**
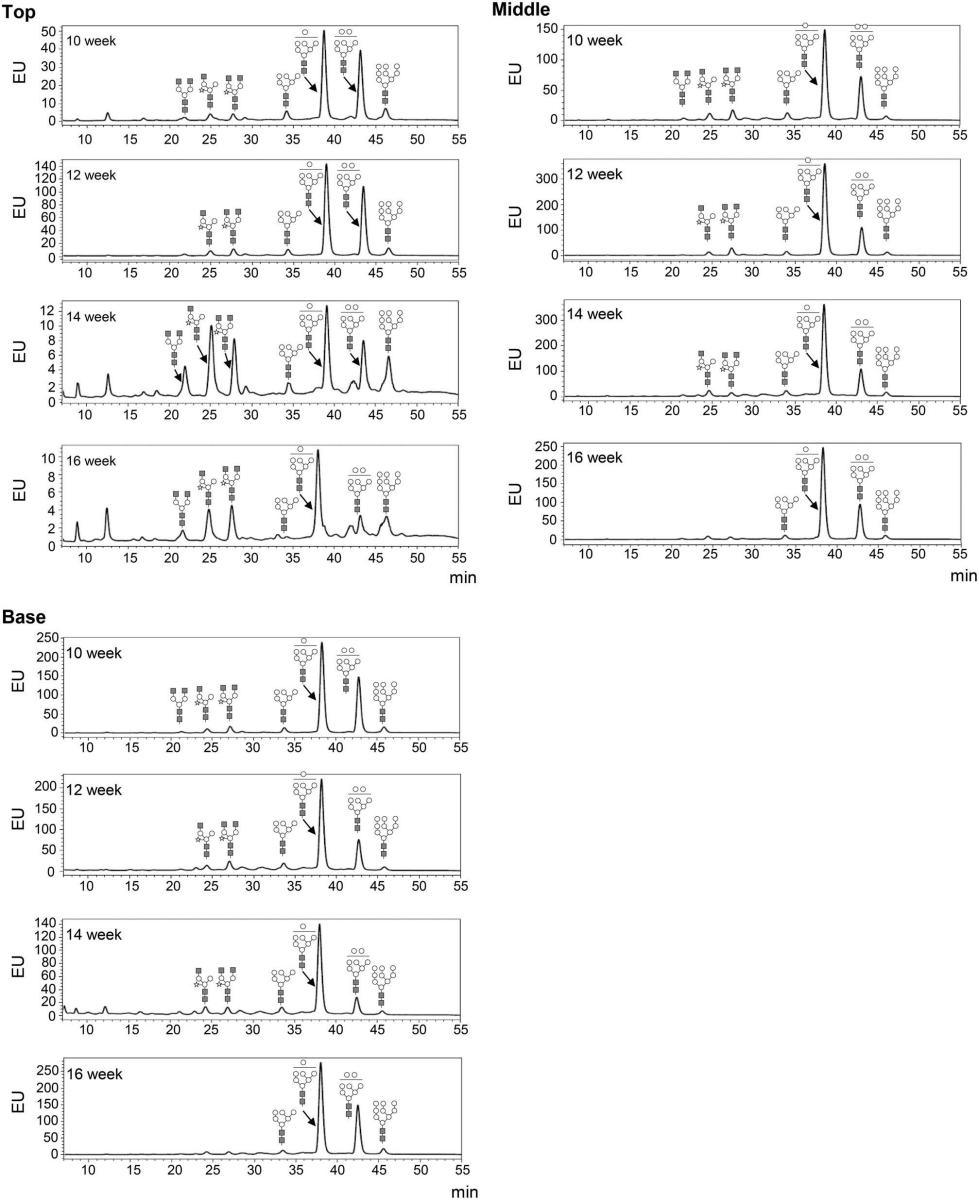
**Glycosylation analysis of GA733-FcK recombinant proteins from leaf tissues at different positions**. The profiles of *N*-glycans released from GA733-FcK were analyzed using HPLC profile of AB-labeled glycans. The proposed glycan structures for each peak are designated. GlcNAc, mannose, and xylose are depicted with black squares, white circles, and white stars, respectively. T, top leaf; M, middle leaf; B, basal leaf.

**Figure 6:**
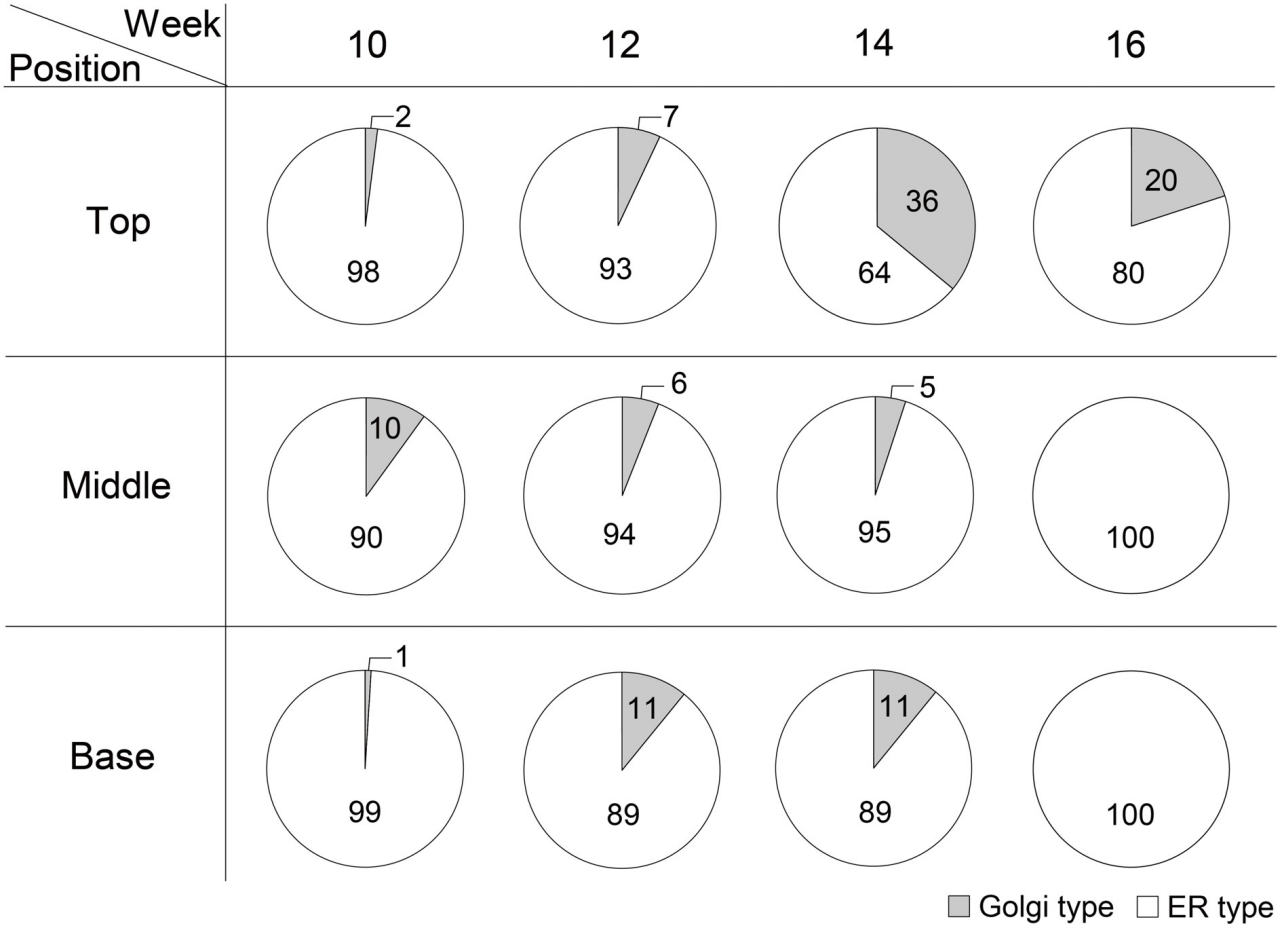
**The ratio of Golgi/ER type glycans of GA733-FcK in top, middle, and basal leaf tissues at 10, 12, 14, and 16 weeks after transplanting**.

**Figure 7:**
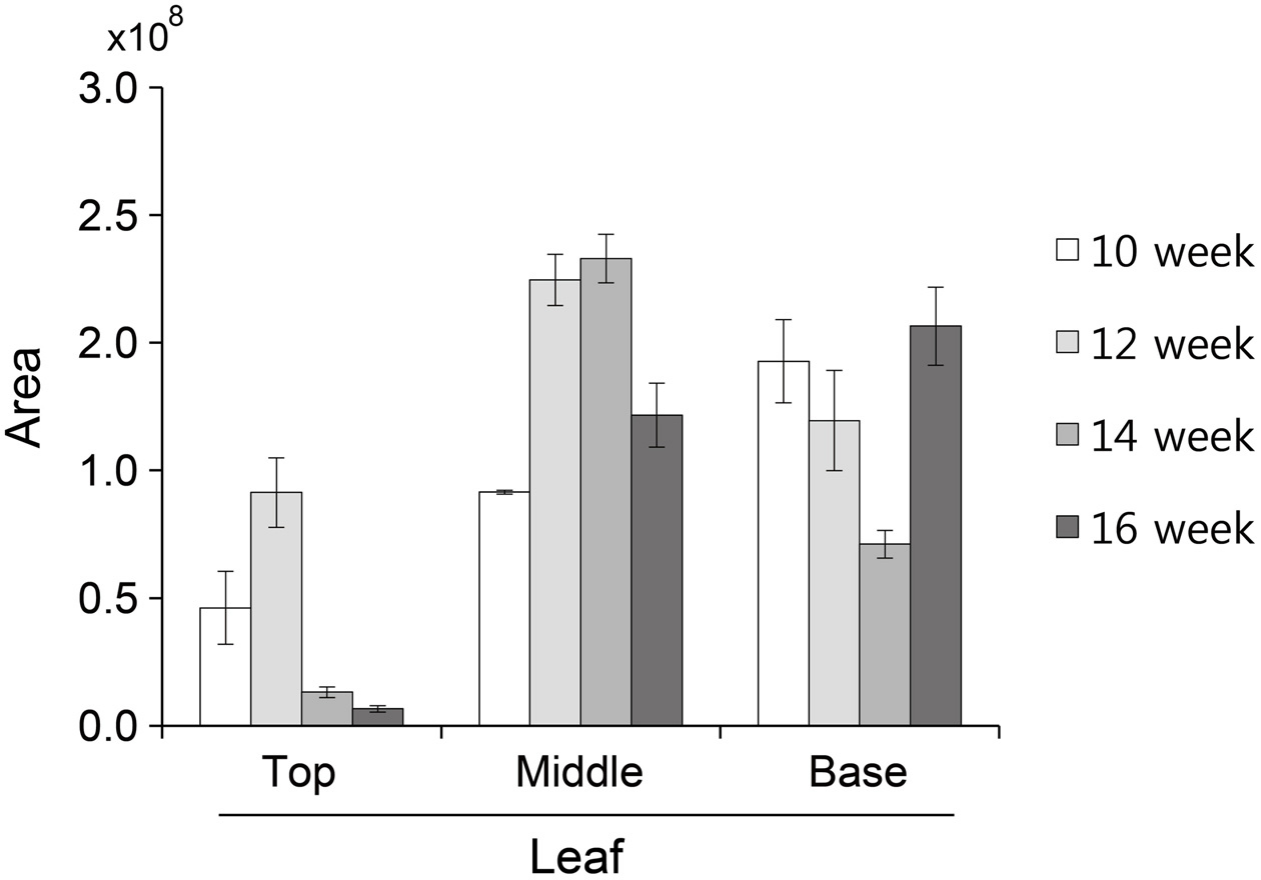
***N*-Glycan quantification of GA733-FcK proteins isolated from top, middle, and basal leaves**. Relative *N*-glycan expression levels of GA733-FcK with aging and at different positions of leaf tissues quantified by HPLC. The area is based on the absolute value of peak intensities in HPLC analysis.

**Figure 8:**
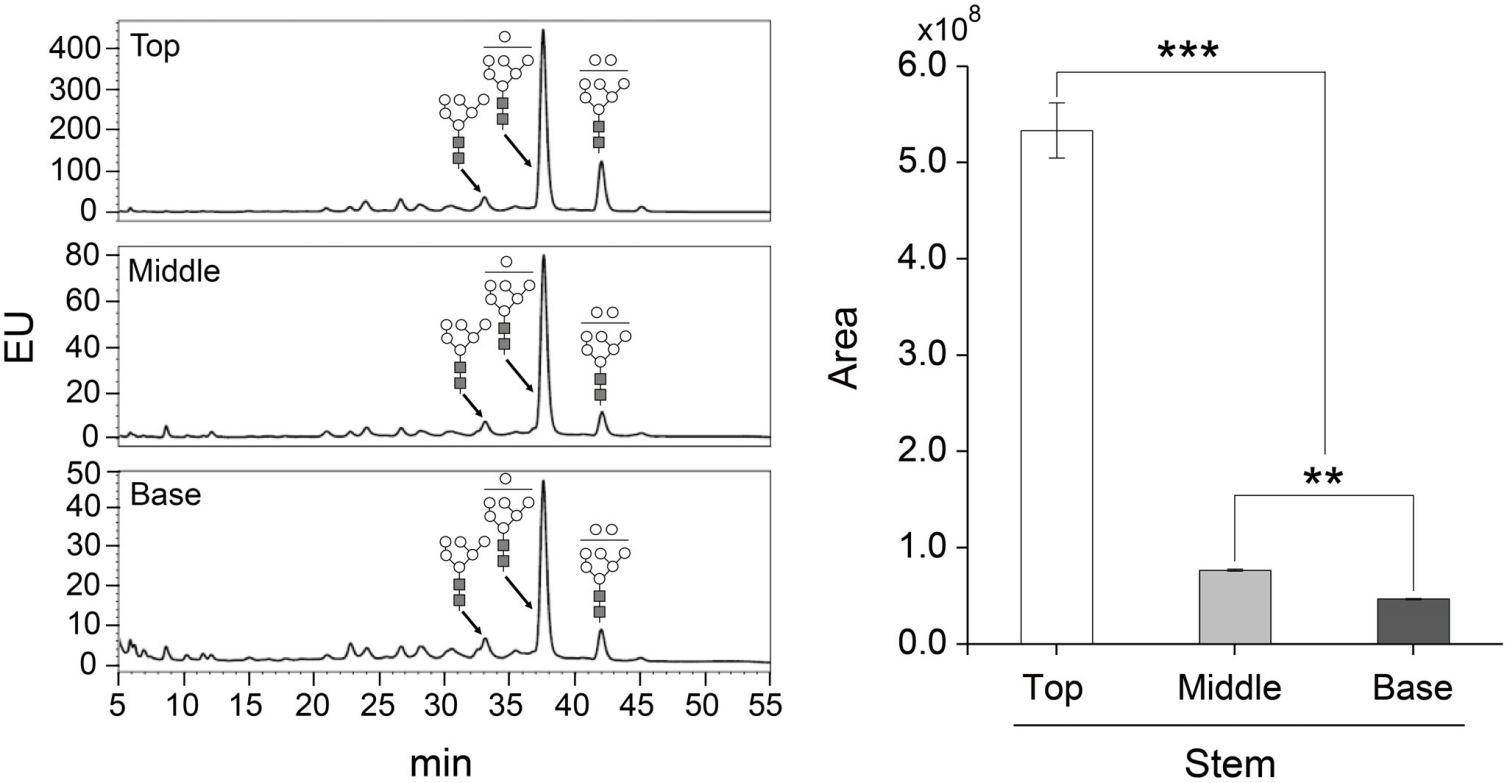
**Glycosylation analysis of GA733-FcK proteins isolated from stem sections (top, middle, and base)**. The profiles of *N*-glycans released from GA733-FcK were analyzed using HPLC profile of AB-labeled glycans. The proposed glycan structures for each peak were designated. GlcNAc and mannose are depicted with black square and white circle, respectively. Relative *N*-glycan expression levels of GA733-FcK at different section of the stem were analyzed by HPLC. The area is based on the absolute value of peak intensities in HPLC analysis. Data represent means and standard errors. Asterisks indicate significant differences (^**^P < 0.05, ^***^P < 0.01) inferred with *t*-test.

**Figure 9:**
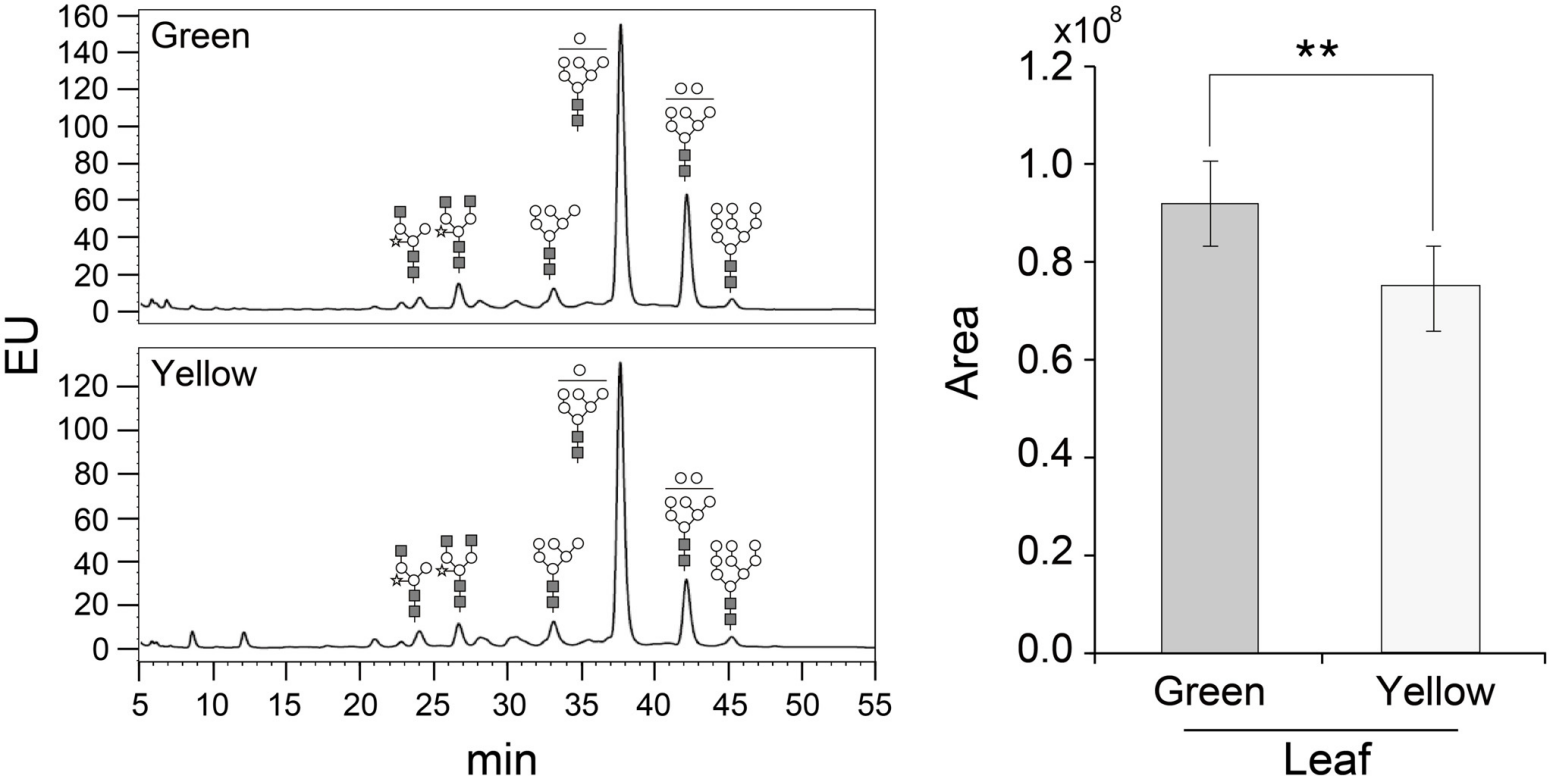
**Glycosylation analysis of GA733-FcK proteins isolated from leaves (yellow and green)**. The profiles of *N*-glycans released from GA733-FcK were quantified using HPLC profile of AB-labeled glycans. Relative *N*-glycosylation levels of GA733-FcK in leaves of different color were analyzed by reverse-phase HPLC. The area is based on the absolute value of peak intensities in HPLC analysis. GlcNAc, mannose, and xylose are depicted with black square, white circle, and white star, respectively. Data represent means and standard errors. Asterisks indicate significant differences (^**^P < 0.05) inferred with *t*-test.

The glycosylation patterns were analyzed in leaves (top, middle, and basal) in relation to the time after transplanting (Figures 5, 6 and Table 1; Supplemental Data Sheet 1). In the top leaves, the plant-specific glycans (Golgi type) suddenly increased at 14 weeks. The relative percentage of Golgi type glycan structures were 36% and 20% in 14 and 16 weeks, respectively. In the middle leaves, the percentage of plant-specific glycan structures (Golgi type) decreased with the time. In the basal leaves, the early growth period (10 weeks) showed the lowest level of plant-specific glycan structures (Golgi type) (1%). Percentage of the plant-specific glycan structures (Golgi type) slightly increased up to 11% until 14 weeks and decreased at 16 weeks. The area of *N*-glycan peaks of GA733-FcK proteins isolated from the top, middle, and basal leaves varied with the growth period (10, 12, 14, and 16 weeks) (Figure 7). The glycan area of proteins isolated from the top leaves was relatively smaller than those of GA733-FcK proteins isolated from the middle and basal leaves. In the top and middle leaves, the area increased until 12 and 14 weeks, respectively, and dramatically decreased thereafter. In contrast, in basal leaves, the glycan area decreased until 14 weeks and increased at 16 weeks. The *N*-glycans of GA733-FcK proteins isolated from the top, middle, and base of the stems of tobacco plants were analyzed by high performance liquid chromatography (HPLC) (Figure 8). All of the three stem sections showed similar high-mannose type glycan structures. However, the glycan area size of the top section of the stems was significantly higher than in both middle and base sections of the stems (*P* < 0.001). The size of the glycan area at the middle stem was slightly higher than at the base section of the stem. In addition, the glycans of GA733-FcK proteins isolated from green and yellow leaves of tobacco plants grown until 16 weeks after transplanting were analyzed by HPLC (Figure 9). The glycan profiles were similar between green and yellow leaves; however, the overall glycan areas of the yellow leaves were slightly smaller than the green leaves.

**Table 1:** **Relative amounts of *N*-glycan structures of GA733-FcK isolated from leaves (top, middle, and basal) of transgenic plants**.

**(week)**	**Top**				**Middle**				**Base**			
	**10**	**12**	**14**	**16**	**10**	**12**	**14**	**16**	**10**	**12**	**14**	**16**
Man9	2	5	10	8	9	3	3	3	4	2	3	4
Man8	26	36	22	7	26	20	19	26	33	20	12	32
Man7	63	48	22	42	53	67	65	69	55	62	66	61
Man6	4	3	3	1	5	3	6	3	3	5	7	3
GlcNAc_2_Man_3_Xyl_1_GlcNAc_2_	2	3	14	18	5	5	3	^*^	2	6	5	^*^
GlcNAc_1_Man_3_Xyl_1_GlcNAc_2_	2	3	20	17	2	1	4	^*^	3	4	6	^*^
GlcNAc_2_Man_3_GlcNAc_2_	1	1	9	7	0.3	^*^	^*^	^*^	0.4	^*^	^*^	^*^

*The amounts are based on relative peak intensities in HPLC and expressed as a percentage of total N-glycans (%)*.

* *Not detectable*.

## Discussion

We demonstrated that the growth stage and tissue position affected the expression of recombinant GA733-FcK glycoprotein and its *N*-glycan structure profile in plants (Chargelegue et al., 2000; Lowe, 2002). The GA733-FcK is a glycoprotein GA733 fused to the Fc fragment of IgG1 type human immunoglobulin, which contain four and one glycosylation sites (Lu et al., 2012). The N-terminus of GA733 was fused with a signal peptide sequence (MATQRRANPSSLHLITVFSLLAAVVSAEVD) from *Nicotiana plumbaginifolia* to be properly targeted into ER, and the C-terminus of the Fc was fused to KDEL, the ER retention signal that marks the proteins to be retained in ER. The constitutive expression of GA733-FcK gene was under the control of a duplicated CaMV 35S promoter (Lu et al., 2012). The F_3_ seedlings of line T303 with the highest expression, which were selected for *in vitro* media, were used for our current experiments. In this study, we hypothesized that the GA733-FcK expression and its *N*-glycan structure varied depending on the location of the plant tissues and the growth age, which influence both the quantity and quality of recombinant proteins expressed in transgenic plants. Thus, the main goal of this study was to elucidate the effect of the growth stage and location of plant leaves and stem tissues (top, middle, and base) on expression of the recombinant protein and its glycosylation, which in turn will help in optimizing the best harvesting time and tissue position for high protein expression and its ideal glycosylation.

For production of highly valuable recombinant therapeutic proteins, tobacco plants have several advantages over other plants such as highly efficient transformation, relatively short period for mass seed production, significant leaf biomass, and easy processing for protein purification due to soft leaf tissue (Daniell et al., 2001; Elbers et al., 2001; Valdes et al., 2003b; Pujol et al., 2005). However, the transgenic plant expression system can be fully realized only when the recombinant protein production level per unit of plant biomass is high and with good quality (Ko and Koprowski, 2005). Thus, the plant harvesting time and tissue positions should be optimized to obtain the highest biomass with good quantity and quality of recombinant proteins. In this study, leaf length and height of the plant were measured to determine the growth and increase in biomass over time. It was found that the growth patterns between transgenic plants and non-transgenic plants did not differ significantly (Figure 1). The plant height and leaf length increased rapidly at 7–8 weeks after transplanting of the *in vitro* seedlings to soil pot in a greenhouse. The plant height continued to increase until 18 weeks, whereas the leaf length growth continuously decreased until 10 weeks (Figure 1). It seems that the leaf length growth slowly decreased after the flowering was initiated at 12 weeks. These results suggest that the best time to harvest to obtain high plant biomass production should be between 12 and 14 weeks for tobacco plants.

The variation in mRNA levels of the recombinant GA733-FcK transgene at different tissue locations during the growth period has not been studied in detail until now. Thus, real-time PCR was applied to investigate whether the mRNA levels of the GA733-FcK gene changed at different tissue positions along the stem (top [T], middle [M], and base [B]) and in leaves harvested at different growth periods (10, 12, 14, and 16 weeks). In the top leaves, the expression ratio increased until 14 weeks and then decreased. This decline in the mRNA levels has been associated with aging and tissue position (Mullet and Klein, 1987). In the middle and basal leaves, the GA733-FcK mRNA levels increased with age, although the increase in the basal leaves was not as rapid as that seen in the middle leaves. These results indicated that the transcription levels of the GA733-FcK gene varied with tissue position and during the plant growth.

It has been reported that recombinant protein levels dramatically change in developmental stage and environmental conditions (Stevens et al., 2000; Elbers et al., 2001; Valdes et al., 2003a; Jamal et al., 2009). Thus, recombinant GA733-FcK protein levels were investigated at different tissue positions during the growth period. Quantitative immunoblot was applied to investigate the change in GA733-FcK protein levels in the top, middle, and basal leaves at 10, 12, 14, and 16 weeks. In the top leaves, the protein level increased until 12 weeks and then decreased. The middle leaves showed the highest GA733-FcK protein level (416 ng/mg FW) compared to the others in terms of overall growth. It is speculated that the reduction of the recombinant GA733-FcK proteins in top leaves is due to energy translocation for flower formation; at this time, biological energy generated in the top leaves is sent to floral meristems. These results are consistent with the previous report of decreased protein expression after flowering (Woodson, 1987). The basal leaves showed stronger non-specific band density compared to the top and middle leaves. These results can be explained by the fact that basal leaves being more senescent produce proteases to degrade recombinant proteins. This is supported by much lower protein levels and less chloroplast in yellow leaves compared with green leaves. The loss of chloroplast means the loss of energy production capacity resulting in reduced protein production capacity (Munne-Bosch and Alegre, 2002). Therefore, these results are not unexpected. In all leaves, the protein level increased until flowering and decreased after that. Proteins degrade at higher rate with age (Woodson, 1987). To study the effect of senescence on protein expression, different expression patterns of the top, middle, and basal stem sections harvested from plants at 18 weeks were analyzed with the densitometry analysis. In stem, protein expression level declined from the top to the base. It is speculated that the bottom section of the stem has less biologically active cells compared to the top and middle stem sections. The low protein expression at the stem base and in yellow leaves is due to their senescence. This study shows that GA733-FcK protein levels at different leaf positions during developmental stage were not harmonized with the mRNA levels; instead, the amount of mRNA was similar to the amount found at 10 weeks. Overall, the highest transcriptional level was observed in the middle leaf at 16 weeks. It is speculated that low protein level is due to reduced translation modification factors such as RNA transport, post-transcriptional regulation, translation, and protein modification process, even at high mRNA levels in plant tissue. Furthermore, proteins degrade after translation due to their senescence. Thus, proteins cannot be expressed with higher levels of mRNA expression (Mullet and Klein, 1987). Therefore, when the plant biomass is harvested for protein purification in downstream processing, yellow leaves should not be included.

Although numerous previous studies have reported that the glycosylation variation is associated with diverse environmental factors including temperature and light conditions, the variations in glycosylation profiles with the developmental stage and tissue positions are not fully understood. Our current results support previous reports correlating the glycan structural differences with aging and tissue positions (Stevens et al., 2000; Elbers et al., 2001; Gleba et al., 2005; Jamal et al., 2009). All leaf and stem samples had mostly oligo-mannose glycan structures, indicating that the KDEL ER retention signal efficiently retained the glycoproteins with oligo-mannose (Ko et al., 2003). In our current study, we were able to confirm the differences in the glycan structure during developmental growth and aging. In the top leaves, plant-specific glycan structure such as β (1,2)-xylose increased between 14 and 16 weeks, whereas in the middle leaf, plant-specific glycan structure decreased with aging. In the basal leaves, the early growth period (10 weeks) showed the lowest level of plant-specific glycan structures (Golgi type). The area of *N*-glycan peaks of GA733-FcK proteins isolated from the top, middle, and basal leaves varied with the growth period (10, 12, 14, and 16 weeks). The glycan peak area from the top leaves was relatively smaller than the middle and basal leaves. Particularly in the top leaves, the area increased until 12 weeks and dramatically decreased afterwards, which was in contrast to the middle leaves where the area increased until 14 weeks and then decreased. However, in basal leaves, the area decreased until 14 weeks and increased at 16 weeks. At 12 weeks, flowers near the top leaves started blooming reaching full bloom at 14 weeks. It is speculated that in plants, development and growth of floral organs require large quantities of sugars from neighboring leaves to generate required energy (Woodson, 1987). Thus, during flowering, physiological changes result in reduction of protein and glycosylation levels along with the increasing ratio Golgi/ER type *N*-glycans in top leaves. This is probably due to the plant focusing its biological activities on development and growth of floral organs. The amount of glycans on proteins was high in the middle and basal leaves, which is consistent with a previous report confirming the accumulation of glycan in plants (Elbers et al., 2001). In addition, changes in glycosylation patterns were analyzed at three stem sections (top, middle, and base). When the plant growth ceased, the glycosylation analysis of proteins extracted from the stem showed no glycan structural difference in all three stem sections. However, the glycan peak areas were quantitatively different among the top, middle, and base sections of the stem. The peak area of the glycosylation amount of stems was smaller than that of leaves. In contrast with the leaves, the glycosylation peak area of the top stem was greater than in the middle and base stem sections. This difference is probably due to the top portion of stems having better sugar supply from leaves compared with the other sections. Protein levels were different between green and yellow leaves, whereas glycosylation level and glycan structure did not significantly differ between green and yellow leaves, which is inconsistent with a previous study showing age dependent glycosylation variation (Elbers et al., 2001). To understand this discrepancy between the current and previous studies, it should be noted that the glycoprotein GA733-FcK bore KDEL ER retention signal, whereas the glycoprotein mouse IgG antibody examined in the previous study (De Boer et al., 1996) did not have KDEL and were secreted through a default secretion pathway. In this study, thus, the ratio Golgi/ER type glycan structures of proteins vary depending on the tendency of leakiness of the glycoproteins.

The post-translational modification of a recombinant vaccine candidate protein is of special importance for its immunogenicity. *N*-Glycosylation of proteins in eukaryotic cells is accomplished in two distinct organelles, the ER and Golgi (Helenius and Aebi, 2004; Ko et al., 2004). The glycan structures have an important effect on therapeutic protein characteristics such as protein folding, *in vivo* half-life, and immunity (Lee et al., 2013). Since the events of *N*-glycan modification in plant differ from those in mammals, recombinant proteins produced in transgenic plants will not be identical to mammalian glycoproteins. However, the folding and assembly of recombinant proteins as well as the transfer of an oligosaccharide precursor to *N*-glycosylation sites can be correctly accomplished in transgenic plant systems (Chen et al., 2005). The xylose and fucose are plant-specific glycan structures that induce an allergic reaction when administered to humans. This problem may be solved by the application of KDEL (ER retention sequence) as the predominant expression system in the production of therapeutic glycoproteins. KDEL (ER retention sequence) attached in the ER to the C-terminal retains a glycoprotein to avoid plant-specific glycan attachment (Ko and Koprowski, 2005; Liu and Howell, 2010; Ruiz-May et al., 2012).

In previous studies (Elbers et al., 2001; Jamal et al., 2013), the protein expression and glycosylation were simply analyzed at only leaf position of plant according to only temperature and light conditions. Previously reported conditions were insufficient to be used to optimize for mass production of the plant-derived recombinant proteins. Although it has been known that the glycosylation variation is associated with environmental factors such as temperature and light conditions, the variations in glycosylation profiles with the developmental stage (before and after flowering) and tissue positions (leaf and stem) have not been fully studied. Our current results are consistent with previous reports related to the glycan structural differences with developmental stage and tissue positions of plants. In this study, also we confirmed that the stem tissues expressed the GA733-FcK with good amount of proteins compared to the leaves. These results suggest stem tissues including leaf tissues can be harvested to purify the recombinant GA733-FcK. In addition, we confirmed that the glycan structures with high mannose were homogeneous at the 12 weeks, but plant specific glycan structures were observed after 14 weeks. These results indicate that the farmers should harvest plants at least before for 12 weeks (floral organ formation period). According to these points, it is considered that the results obtained in this study are meaningful information for commercialization of GA733-FcK producing plants.

In this study, the effect of aging and position of tissues was identified by gene expression and glycosylation pattern. The results described herein represent an overview of the effect of aging and position of tissues that should be considered prior to use of the plant-derived antigen in the large-scale production of vaccine and harvesting. Taken together, we demonstrated the variation in glycan structures and levels of recombinant antigen antibody fusion protein GA733-FcK during flowering. This is the first report of the ratio Golgi/ER type glycans of recombinant glycoproteins increase in young leaves during flowering. The correlation between glycosylation and energy dynamics during flowering in plants warrants further detailed research.

### Conflict of interest statement

The authors declare that the research was conducted in the absence of any commercial or financial relationships that could be construed as a potential conflict of interest.
