# Supplementary material for: Effect of the developmental stage and tissue position on the expression and glycosylation of recombinant glycoprotein GA733-FcK in transgenic plants
Source: Front Plant Sci. 2015 Jan 13;5:778. doi: 10.3389/fpls.2014.00778 (PMC4292234; doi:10.3389/fpls.2014.00778)
Supplement: Supplementary file 1 [file DataSheet1.DOCX]

***Supplementary Material***

**Effect of the developmental stage and tissue position on the expression and glycosylation of recombinant glycoprotein GA733-FcK in transgenic plants**

**Chae-Yeon Lim**^1^, **Kyung Jin Lee**^1^, **Doo-Byoung Oh**^2^ and **Kisung Ko**^1*^

^1^Department of Medicine, Medical Research Institute, College of Medicine, Chung-Ang University, Seoul, Korea

^2^Korea Research Institute of Bioscience & Biotechnology (KRIBB), 125 Gwahakro, Yuseong-gu, Daejeon 305-806, Korea

***Correspondence**: Kisung Ko, Department of Medicine, Medical Research Institute, College of Medicine, Chung-Ang, University, Seoul 156-756 Korea; Email: [ksko@cau.ac.kr](mailto:ksko@cau.ac.kr)

1. **Supplementary Data**

Purified glycans were dried and then re-dissolved in a mixture of 90 μL dimethyl sulfoxide (DMSO), 2.7 μL water, and 35 μL iodomethane for solid phase permethylation using a spin column method (Goetz et al., 2009). The chloroform layer containing permethylated glycans was dried and resuspended in 4 μL of 50% methanol solution, and mixed in equal volumes with the matrix 2,5-dihydroxybenzoic acid prepared in 1 mM sodium acetate solution. The resulting mixture was applied onto a MALDI MSP 96 polished steel Chip (Bruker Daltonik GmbH, Bremen, Germany) and dried. MALDI-TOF mass spectrometry was performed in the reflector positive ion mode using a Microflex (Bruker Daltonik). All mass spectra were acquired at 20-kV accelerating voltage using the method recommended by the manufacturer.

1. **Supplementary Figures**

## Supplementary Figure


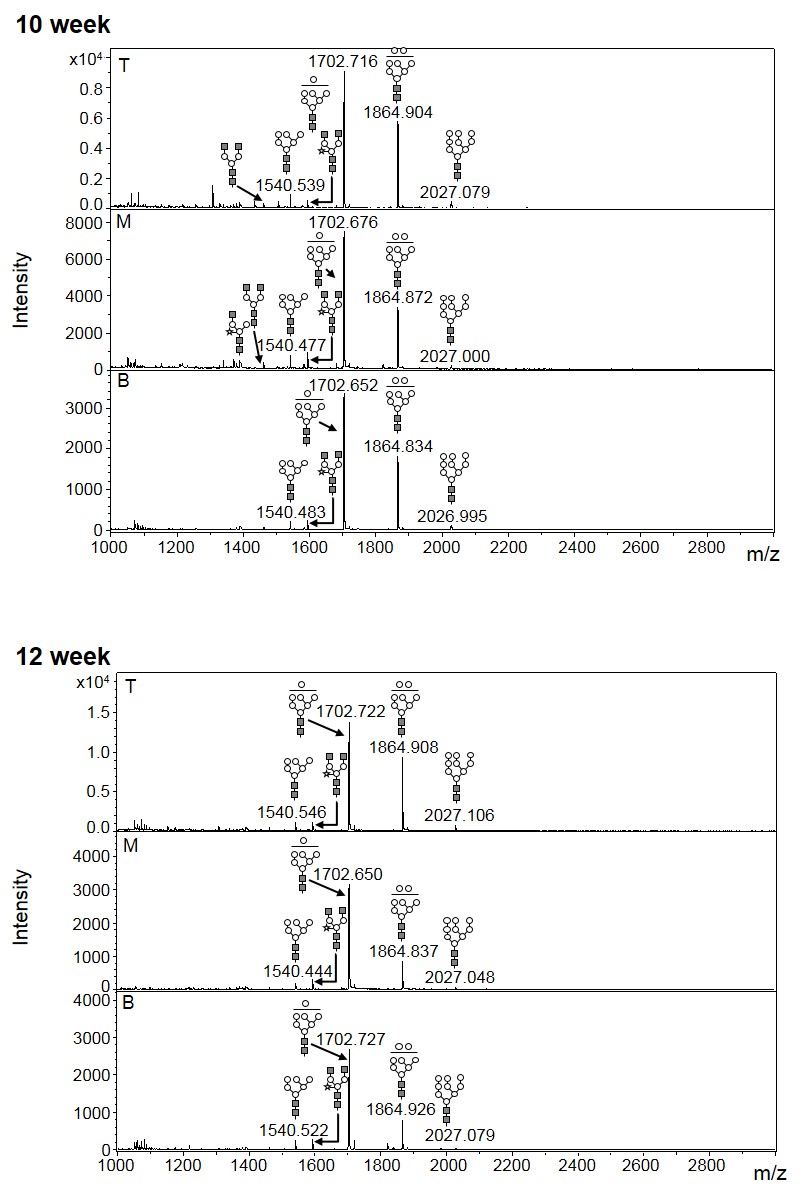

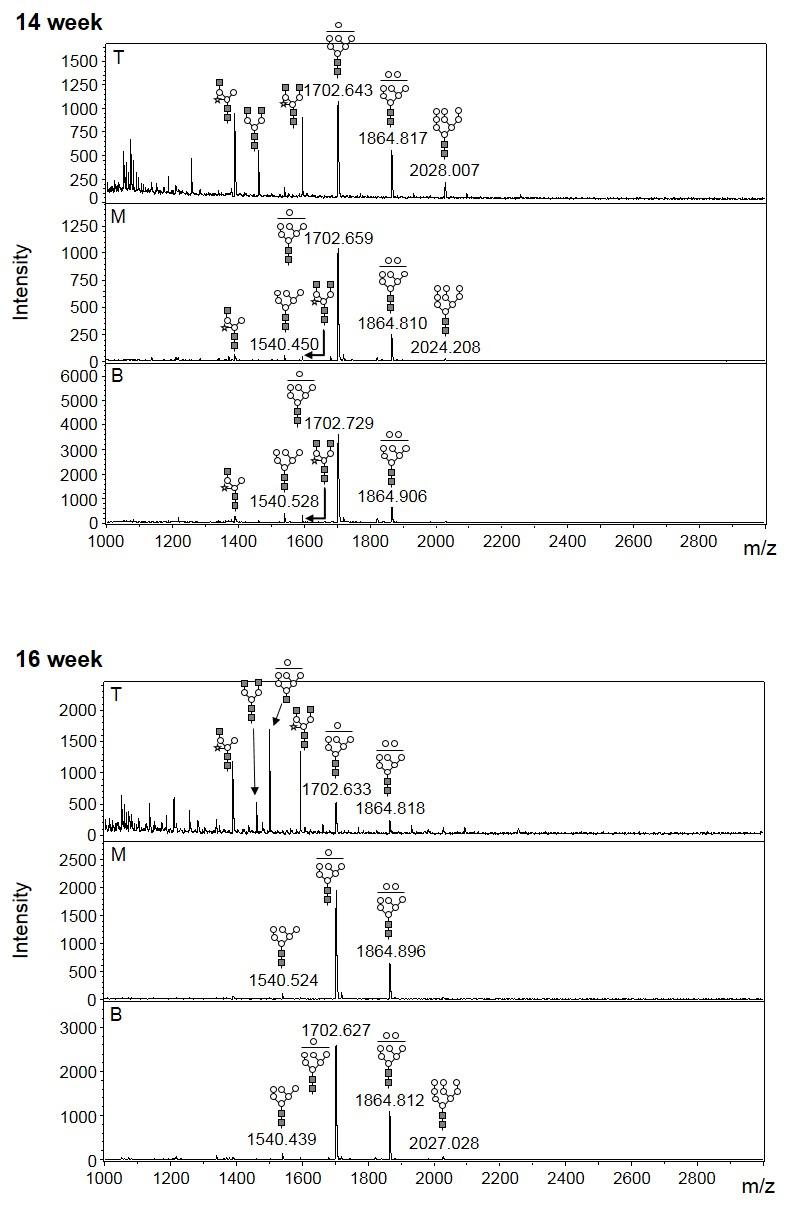


**Supplementary 1** The profiles of N-glycans released from GA733-FcK were analyzed using MALDI-TOF mass spectrometry after permethylation.

1. **Reference**

Goetz, J. A., Novotny, M. V., and Mechref, Y. (2009). Enzymatic/chemical release of O-glycans allowing MS analysis at high sensitivity. *Anal Chem* 81**,** 9546-9552. doi: 10.1021/ac901363h.
