# Supplementary material for: Effect of the developmental stage and tissue position on the expression and glycosylation of recombinant glycoprotein GA733-FcK in transgenic plants
Source: Front Plant Sci. 2015 Jan 13;5:778. doi: 10.3389/fpls.2014.00778 (PMC4292234; doi:10.3389/fpls.2014.00778)
Supplement: Supplementary file 2 [file DataSheet2.DOCX]

***Supplementary Material***

**Effect of the developmental stage and tissue position on the expression and glycosylation of recombinant glycoprotein GA733-FcK in transgenic plants**

**Chae-Yeon Lim**^1^, **Kyung Jin Lee**^1^, **Doo-Byoung Oh**^2^ and **Kisung Ko**^1*^

^1^Department of Medicine, Medical Research Institute, College of Medicine, Chung-Ang University, Seoul, Korea

^2^Korea Research Institute of Bioscience & Biotechnology (KRIBB), 125 Gwahakro, Yuseong-gu, Daejeon 305-806, Korea

***Correspondence**: Kisung Ko, Department of Medicine, Medical Research Institute, College of Medicine, Chung-Ang, University, Seoul 156-756 Korea; Email: [ksko@cau.ac.kr](mailto:ksko@cau.ac.kr)

1. **Supplementary Data**

Equal amount of the protein between mammalian-derived GA733-Fc and plant-derived GA733-FcK with their purity was confirmed via the SDS-PAGE. These data were not shown in this manuscript.

1. **Supplementary Figures**

## Supplementary Figure


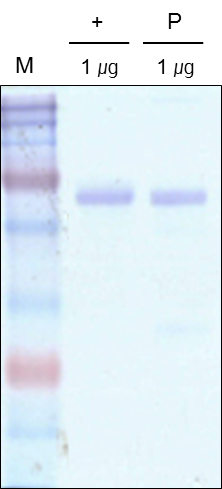


**Supplementary 2** M: protein marker; +: positive control (mammalian derived GA733-Fc); P: purified plant-derived GA733-FcK.
