# Supplementary material for: Effect of the developmental stage and tissue position on the expression and glycosylation of recombinant glycoprotein GA733-FcK in transgenic plants
Source: Front Plant Sci. 2015 Jan 13;5:778. doi: 10.3389/fpls.2014.00778 (PMC4292234; doi:10.3389/fpls.2014.00778)
Supplement: Supplementary file 3 [file DataSheet3.DOCX]

***Supplementary Material***

**Effect of the developmental stage and tissue position on the expression and glycosylation of recombinant glycoprotein GA733-FcK in transgenic plants**

**Chae-Yeon Lim**^1^, **Kyung Jin Lee**^1^, **Doo-Byoung Oh**^2^ and **Kisung Ko**^1*^

^1^Department of Medicine, Medical Research Institute, College of Medicine, Chung-Ang University, Seoul, Korea

^2^Korea Research Institute of Bioscience & Biotechnology (KRIBB), 125 Gwahakro, Yuseong-gu, Daejeon 305-806, Korea

***Correspondence**: Kisung Ko, Department of Medicine, Medical Research Institute, College of Medicine, Chung-Ang, University, Seoul 156-756 Korea; Email: [ksko@cau.ac.kr](mailto:ksko@cau.ac.kr)

1. **Supplementary Data**

The detected protein bands were digitized to an electronic image, and the band intensity was measured using Image J software (National Institutes of Health, Bethesda, MD). To quantify [ng/(mg of fresh leaf)] the amount of GA733-FcK in transgenic plants, the positive control (31.25, 62.5, 125, 250, and 500 ng) were loaded onto one SDS-PAGE gel. The intensity of each sample and standard was adjusted by subtracting background intensity from the measured intensity. A standard curve between the amount (ng) of the positive control and the measured image intensity of the standard was calculated using Image J software (National Institutes of Health, Bethesda, MD). The intensity value was used to estimate the amount of GA733-FcK proteins (ng/mg) from the linear regression equation derived from the standards.

1. **Supplementary Figures**

## Supplementary Figure


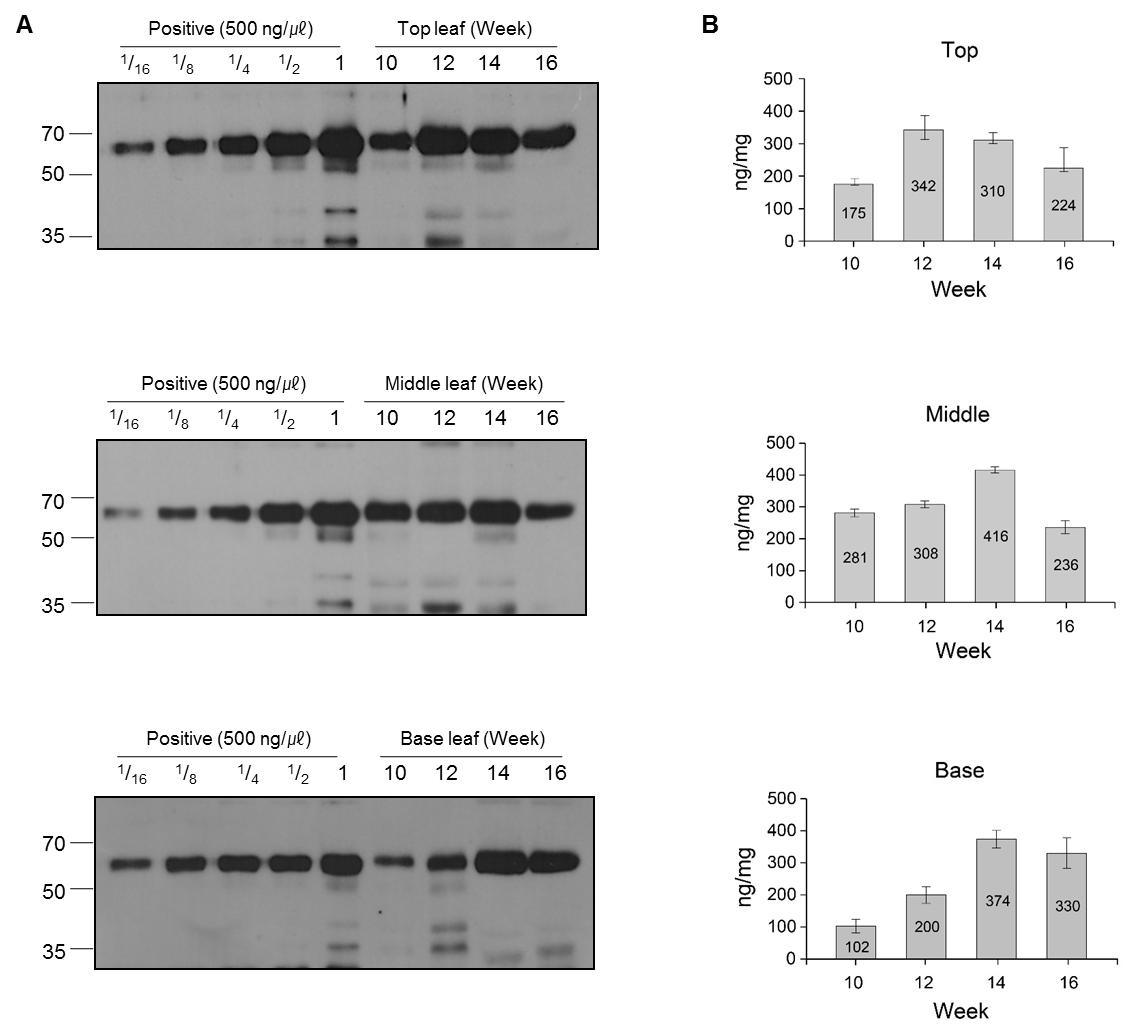


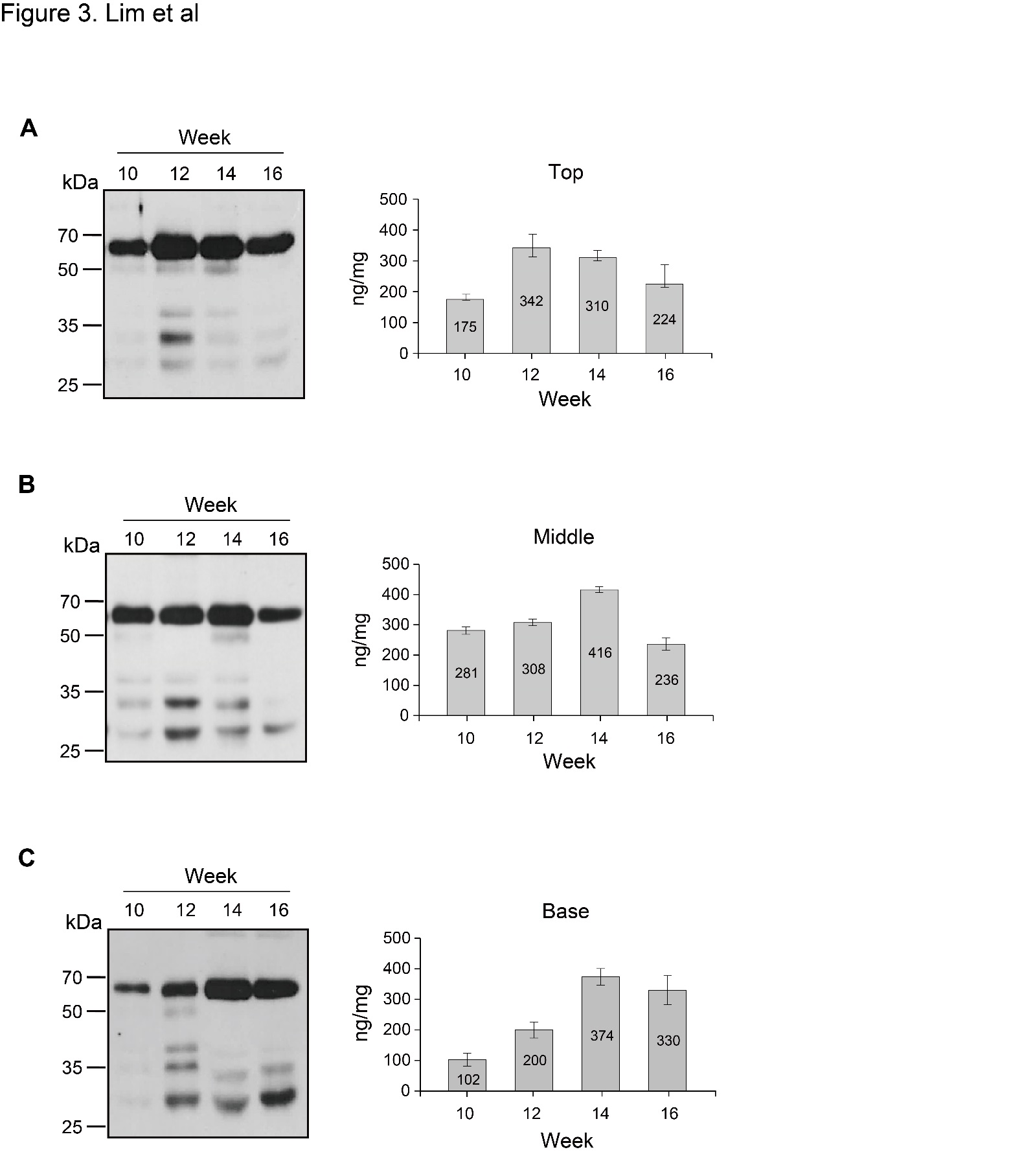

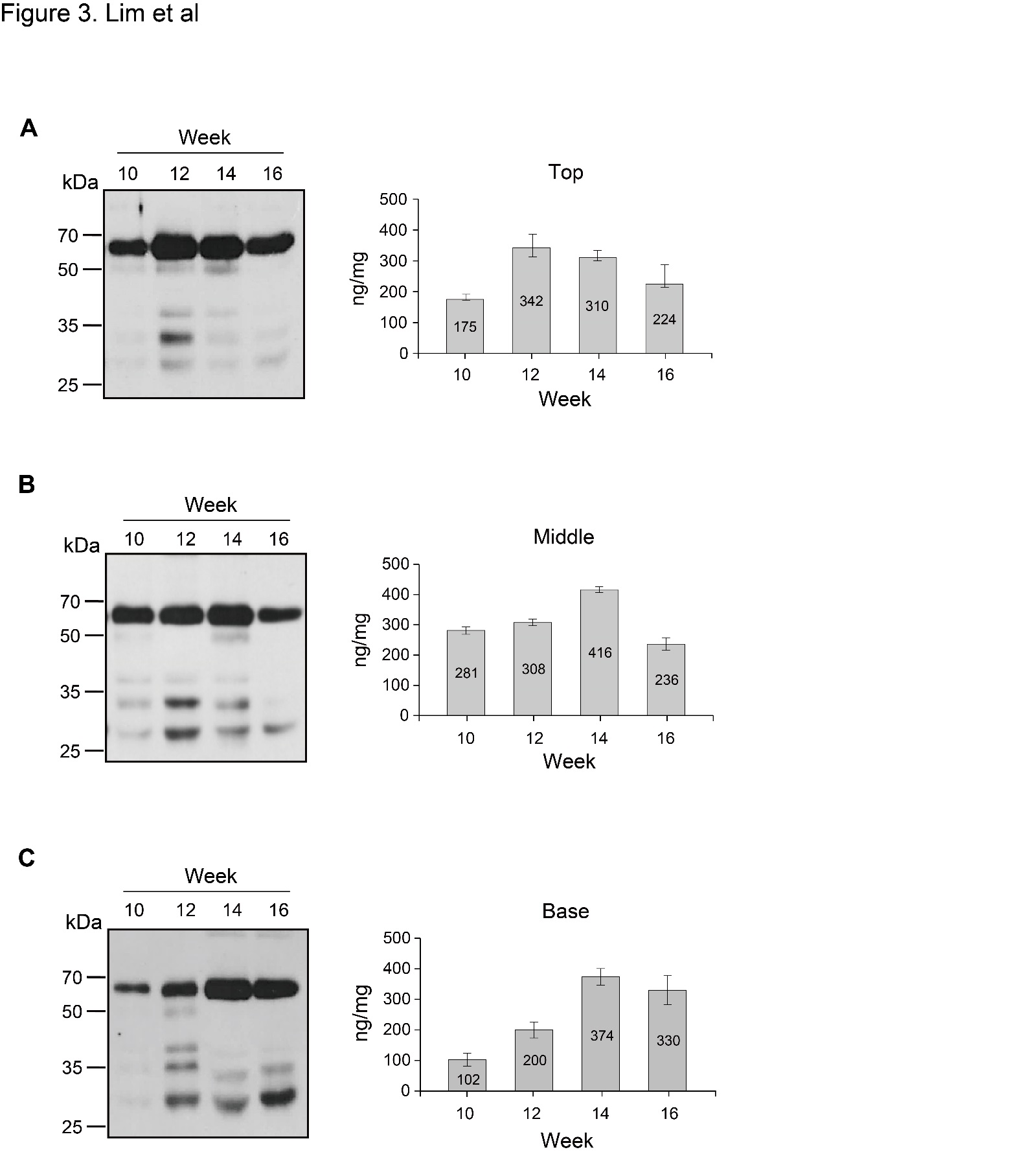

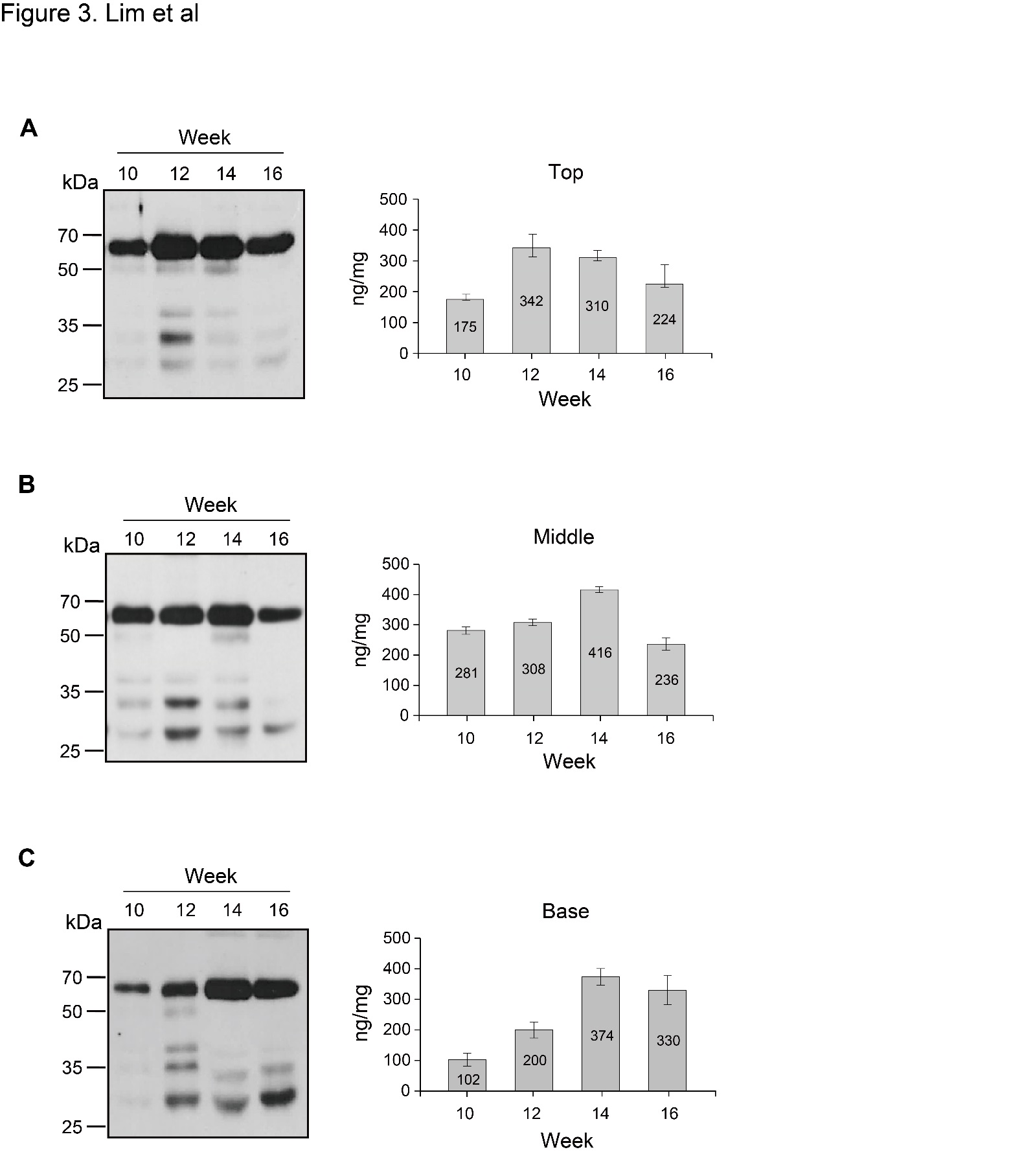
**Supplementary 3** (A) Western blot of the top middle, and base leaf expression. Leaf samples from GA733-FcK transgenic plants were ground with extraction buffer to confirm the protein expression levels. The GA733-FcK proteins were detected by mouse anti-human Fcγ antibody. (B) The graph shows relative quantitation. Positive control; purified of GA733-FcK.

**B**
